# Supplementary material for: Ferritin Heavy Chain Binds Peroxiredoxin 6 and Inhibits Cell Proliferation and Migration
Source: Int J Mol Sci. 2022 Oct 26;23(21):12987. doi: 10.3390/ijms232112987 (PMC9654362; doi:10.3390/ijms232112987)
Supplement: Supplementary file 1 [file ijms-23-12987-s001.zip › ijms-1946872-supplementary.pdf]

Supplementary Materials

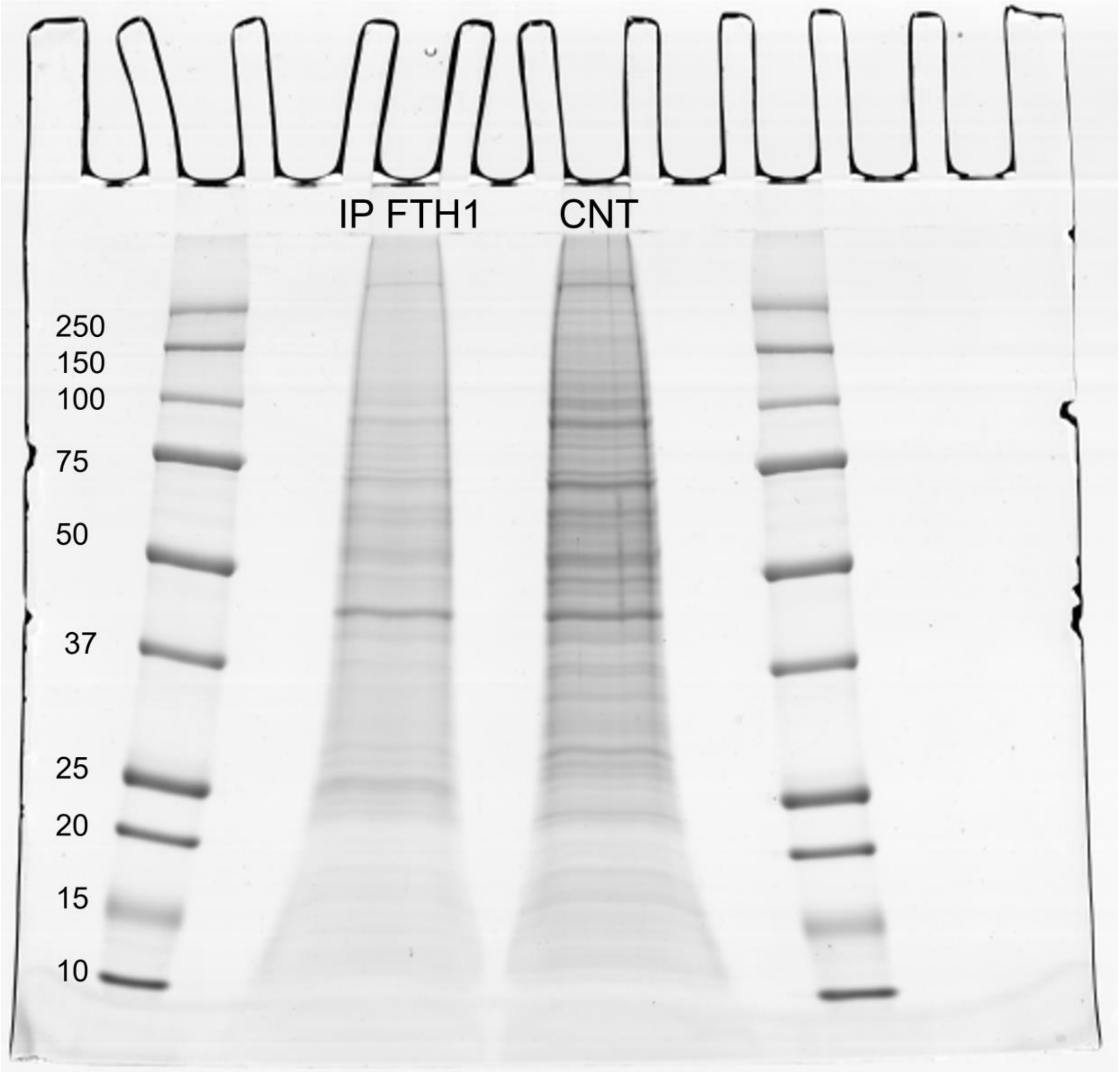

Figure S1 SDS-PAGE of the eluted proteins from the immunoprecipitation experiment (IP FTH1) and control (CNT).

Table S1. List of FTH1 interactors identified in the functional proteomics experiment. Experimental electrophoretic mobility (mass range), protein and gene names, UniProt entry codes and the number of identified peptides (in the bracket those overcoming Mascot threshold) are reported.

Mass Ranges (KDa)	Protein Name	Gene	UniProt Code	Peptides
250-150	Pericentriolar material 1 protein	PCM1	Q15154	7(3)
150-100	Structural maintenance of chromosomes protein 3	SMC3	Q9UQE7	13(8)
	Cytospin-A	SPECC1L	Q69YQ0	7(3)
	Myosin phosphatase Rho-interacting protein	MPRIP	Q6WCQ1	13(6)
	Unconventional myosin-Id	MYO1D	O94832	17(9)
	HAUS augmin-like complex subunit 6	HAUS6	Q7Z4H7	4(3)
100-75	Neurabin-2	PPP1R9B	Q96SB3	10 (6)
	Bromodomain-containing protein 2	BRD2	P25440	3(2)
	Hepatocyte growth factor-regulated tyrosine kinase substrate	HGS	O14964	4(5)
	Nuclear autoantigenic sperm protein	NASP	P49321	5(5)
	Plakophilin-1	PKP1	Q13835	6(4)
	DNA replication licensing factor MCM5	MCM5	P33992	6(3)
	Junction plakoglobin	JUP	P14923	21(12)
	SUN domain-containing protein 2	SUN2	Q9UH99	4(2)
	Protein-glutamine gamma-glutamyltransferase E	TGM3	Q08188	4(4)
75-50	Sec1 family domain-containing protein 1	SCFD1	Q8WVM8	9(8)
	Phosphoenolpyruvate carboxykinase [GTP], mitochondrial	PCK2	Q16822	6(4)
	Nuclear receptor coactivator 4	NCOA4	Q13772	5(2)
	Leukotriene A-4 hydrolase	LTA4H	P09960	4(3)
	Tyrosine-protein phosphatase non-receptor type 11	PTPN11	Q06124	14(7)
	Threonylcarbamoyladenosine tRNA methylthiotransferase	CDKAL1	Q5VV42	3(2)
	Keratinocyte proline-rich protein	KPRP	Q5T749	5(4)
	SNW domain-containing protein 1	SNW1	Q13573	5(4)
	Tyrosine-protein kinase Yes	YES1	P07947	9(5)
	Suprabasin	SBSN	Q6UWP8	4(4)
	Regulator of nonsense transcripts 3B	UPF3B	Q9BZI7	5(3)
	Centrosomal protein of 55 kDa	CEP55	Q53EZ4	10(4)
	Vimentin	VIM	P08670	30(22)
	Peptidyl-prolyl cis-trans isomerase FKBP5	FKBP5	Q13451	7(5)
	WAS protein family homolog 2	WASH2P	Q6VEQ5	2(2)

50-37	Flotillin-1	FLOT1	O75955	2(2)
	Guanine nucleotide-binding protein subunit alpha-13	GNA13	Q14344	2(2)
	Endoplasmic reticulum-Golgi intermediate compartment protein 2	ERGIC2	Q96RQ1	2(2)
	NIF3-like protein 1	NIF3L1	Q9GZT8	2(2)
	cAMP-dependent protein kinase catalytic subunit alpha	PRKACA	P17612	4(2)
	Guanine nucleotide-binding protein G(i) subunit alpha-1	GNAI1	P63096	3(2)
	IST1 homolog	IST1	P53990	3(3)
	Inhibitor of nuclear factor kappa-B kinase-interacting protein	IKBIP	Q70UQ0	5(3)
37-25	Reticulocalbin-2	RCN2	Q14257	8(7)
	Four and a half LIM domains protein 1	FHL1	Q13642	2(2)
	EF-hand domain-containing protein D1	EFHD1	Q9BUP0	6(5)
	Ribosomal RNA small subunit methyltransferase NEP1	EMG1	Q92979	2(2)
	40S ribosomal protein S3	RPS3	P23396	7(3)
	Peroxiredoxin-6	PRDX6	P30041	13(11)
25-20	Heat shock protein beta-1	HSPB1	P04792	8(5)
	Ras-related C3 botulinum toxin substrate 1	RAC1	P63000	5(4)
	Ferritin heavy chain	FTH1	P02794	18(13)
20-10	Ezrin	N/A	Q9UK20	2(2)
	40S ribosomal protein S10	RPS10	P46783	5(3)
	Galectin-7	LGALS7	P47929	6(3)
	Replication protein A 14 kDa subunit	RPA3	P35244	4(2)

Table S2: Co-expression of FTH1 and PRDX6 in different tissue

tissue	description	strength	FDR
BTO:0000570	Hematopoietic system	0,67	0,0017
BTO:0000089	Blood	0,81	0,00084
BTO:0000345	Digestive gland	0,61	0,0135
BTO:0000511	Gastrointestinal tract	0,71	0,0221
BTO:0000759	Liver	0,66	0,0414

Table S3: FTH1 and PRDX6 partner in several biological processes

See excel file Table S3
